# Ischemic stroke reduces bone perfusion and alters osteovascular structure

**DOI:** 10.1016/j.bonr.2025.101824

**Published:** 2025-01-04

**Authors:** Nicholas J. Hanne, Andrew J. Steward, Carla Geeroms, Elizabeth D. Easter, Hannah T. Gensch, Greet Kerckhofs, Tatjana N. Parac-Vogt, Huaxin Sheng, Jacqueline H. Cole

**Affiliations:** a Joint Department of Biomedical Engineering, University of North Carolina, Chapel Hill, NC, and North Carolina State University, Raleigh, NC, USA; b Prometheus, Division of Skeletal Tissue Engineering, KU Leuven, Leuven, Belgium; c Materials Science and Engineering, North Carolina State University, Raleigh, NC, USA; d Institute of Mechanics, Materials and Civil Engineering, UC Louvain, Louvain-la-Neuve, Belgium; e Materials Engineering, KU Leuven, Leuven, Belgium; f Department of Chemistry, KU Leuven, Leuven, Belgium; g Department of Anesthesiology, Duke University Medical Center, Durham, NC, USA

**Keywords:** Ischemic stroke, Bone vasculature, Intraosseous perfusion

## Abstract

Stroke patients lose bone mass and experience fracture at an elevated rate. Although functional intraosseous vasculature is necessary for skeletal maintenance, the effect of stroke on osteovasculature is unknown. In this study we characterized changes to osteovascular perfusion, structure, and composition following mild-to-moderate stroke severity in mice, both with and without exercise therapy. Twelve-week-old male mice (*n* = 27) received either an ischemic stroke (middle cerebral artery occlusion) or sham procedure, followed by a four-week recovery with either moderate daily treadmill or sedentary activity. Intraosseous perfusion, measured weekly in the proximal tibial metaphysis with laser Doppler flowmetry, was reduced for two weeks in the stroke group relative to the sham group. After four weeks, osteovascular structure was assessed in the distal femoral metaphysis with contrast-enhanced computed tomography. Increased osteovascular volume and branching, decreased number of smaller vessels (6–22 μm), and increased number of larger vessels (>66 μm) were observed in the stroke groups compared to sham groups, which may be a compensatory response to early perfusion deficits. Although moderate exercise mitigated the impact of stroke on osteovascular perfusion and volume, it tended to reduce the amount of osteogenic type H vasculature quantified with immunofluorescence microscopy and, exacerbated by stroke effects, produced fewer vessels in close proximity to bone and thus may have detrimental effects on bone remodeling during early stroke recovery. Since results were similar in both limbs, the effects of ischemic stroke on osteovascular perfusion and structure were primarily systemic, rather than resulting from paresis or disuse, providing new insight for future studies on the pathogenesis and treatment of skeletal fragility in stroke patients.

## Highlights

• First study to examine changes to osteovasculature following ischemic stroke
• Intraosseous perfusion decreased during early stroke recovery
• Increased osteovascular volume and branching observed post-stroke
• Fewer osteogenic small vessels near bone surfaces observed with exercise post-stroke

## Abbreviations

Ad.D: Adipocyte density
Ad.Di: Adipocyte diameter
Ad.V: Adipocyte volume
Ma.V: Marrow volume
EMCN: Endomucin
LDF: Laser Doppler flowmetry
Ves.D: Blood vessel density
Ves.Th: Blood vessel thickness
Ves.S-BS: Vessel surface to bone surface distance
Ves.V: Vessel volume

## 1 Introduction

Stroke is the leading cause of disability in the United States and one of the leading causes of disability worldwide (Benjamin et al., 2019). Stroke not only causes cognitive and motor impairments but also negatively affects skeletal health (Kanis et al., 2001; Jørgensen et al., 2000; Jørgensen and Jacobsen, 2001; Ramnemark et al., 1998, Ramnemark et al., 1999; Fisher et al., 2013; Beaupré and Lew, 2006). Stroke patients lose bone mass at an accelerated rate, fall more, and experience hip fracture 2–4 times more frequently than individuals of the same age who have not experienced stroke (Kanis et al., 2001; Jørgensen et al., 2000; Ramnemark et al., 1998). Bone mineral density (BMD) loss and fracture preferentially occur in the paretic limbs of stroke patients (Ramnemark et al., 1999; Fisher et al., 2013). Since bone adapts to altered mechanical loading (Frost, 2003), in particular losing BMD and becoming more susceptible to fracture with unloading (Leblanc et al., 1990; Vico et al., 2000), the current paradigm is that hemiplegia and limb disuse from bedrest drive the skeletal deficits post-stroke (Jørgensen and Jacobsen, 2001; Ramnemark et al., 1999; Beaupré and Lew, 2006). In addition, rehabilitation activity and exercise are correlated with reduced fracture risk and improved skeletal health outcomes following stroke (Jørgensen et al., 2000; Eng et al., 2008; Pang and Lau, 2010; Peppen et al., 2004; Borschmann et al., 2015), further supporting loading as a primary driver. However, a study in severe stroke patients showed lower BMD in various sites on the stroke-affected side, relative to the unaffected side, despite complete bedrest (Beaupré and Lew, 2006). Even after controlling for activity level and other factors, lower vascular elasticity, a metric of vascular health, has been associated with lower polar stress-strain index, a surrogate measure of bone torsional stiffness and strength (Weatherholt et al., 2015), in the radial diaphysis (Pang et al., 2013). Together these results suggest that the negative effects of stroke on bone extend beyond just mechanical unloading and that vascular deficits may also contribute.

Because vasculature is critically important for maintaining functional bone tissue, supplying not only oxygen and nutrients but also cell signaling factors (Stegen and Carmeliet, 2018), vascular dysfunction may contribute to the increased fracture risk experienced by stroke patients. Little is known about the effects of stroke on limb vasculature, particularly on vascular perfusion and structure, and no previous study has examined the effects on the vasculature within bone, or *osteovasculature*. Two previous studies reported decreased blood flow in the affected lower leg compared to the unaffected leg in elderly chronic stroke patients with mild-to-moderate gait asymmetry at least 6 months after moderate ischemic stroke (Ivey et al., 2004, Ivey et al., 2010). Changes to limb blood flow, however, may not be indicative of changes to osteovasculature, which has more direct impacts on bone remodeling and the marrow microenvironment – in osteoporotic individuals, increased intraosseous perfusion measured by contrast-enhanced clinical imaging was strongly correlated with increased bone formation rate in the iliac crest (Reeve et al., 1988) and weakly correlated with higher BMD in the proximal femur (Griffith et al., 2008). In addition, an increased number of capillaries have been observed within 50 μm of bone surfaces at active remodeling sites, compared to non-remodeling bone surfaces, in both humans (Kristensen et al., 2013) and rats (Prisby et al., 2011). The composition of endothelial cells within long bones has been shown to regulate osteogenesis in mice, with endothelial cells that express both endomucin (EMCN) and CD31 (defined as *type H cells*) promoting osteoprogenitor and endothelial cell proliferation and increasing bone formation rate through Notch activity (Ramasamy et al., 2014; Kusumbe et al., 2014; Owen-Woods and Kusumbe, 2022). The effects of stroke on these osteovascular metrics (perfusion, structure, and cellular composition) are unknown and may provide insight into the relationship between bone vasculature and bone loss following stroke. Although characterizing osteovasculature in humans is difficult, it can be examined in animal models, such as the established rodent models of ischemic stroke involving middle cerebral artery occlusion (MCAo) (Koizumi et al., 1986; Longa et al., 1989).

The effect of ischemic stroke on adipose within bone is also unknown. Adipose is a highly vascularized tissue that produces angiogenic and inflammatory paracrine signaling molecules which may interfere with physiologic blood supply to bone and bone-vascular crosstalk (Crandall et al., 1997; Laroche, 2002; Jones, 1993). The effects of marrow adipose on osteovasculature are not well understood, but marrow adipose tissue has been negatively correlated with intraosseous perfusion in the human spine (Griffith et al., 2005, Griffith et al., 2006; Biffar et al., 2010), and exercise reduced the amount of marrow adipose in obese mice (Styner et al., 2014, Styner et al., 2017; McCabe et al., 2019). Examining the changes to adipose tissue within bone may provide insight into the changes to bone and osteovascular health following ischemic stroke with or without exercise.

In our previous study using an MCAo mouse model, we showed that although stroke did not cause detrimental changes to bone microstructure, it prevented the exercise-induced microstructural gains observed in the sham group, even without limb disuse or changes to gait (Hanne et al., 2019a), which directly challenges the current paradigm that bedrest and disuse drive bone changes following stroke. Since bone and vascular health are tightly coupled, we hypothesized that stroke negatively impacts osteovasculature and inhibits the positive effects of treadmill exercise. In this study, we used the same bones to characterize changes to in vivo intraosseous perfusion, blood vessel network microstructure, and endothelial cellular composition following ischemic stroke.

## 2 Methods

### 2.1 Study design

**Fig. 1:**
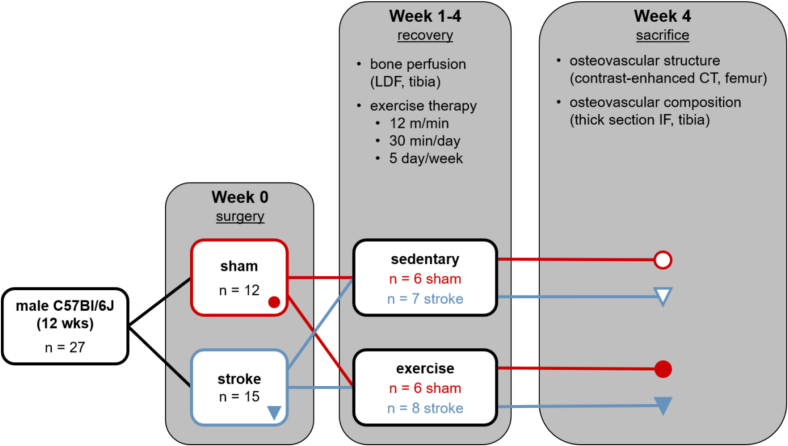
Study design. Mice received stroke or sham surgery, followed by four weeks of treadmill or sedentary activity. Intraosseous perfusion was measured weekly during recovery in affected and unaffected tibiae with laser Doppler flowmetry (LDF). After sacrifice, osteovascular structure and composition were characterized with contrast-enhanced computed tomography (CT) and immunofluorescence (IF) microscopy.

All procedures were approved by the North Carolina State University Animal Care and Use Committee. Animals were group housed 4–5 per cage with a 12-hour diurnal light cycle and free access to standard chow and water. Twenty-seven male C57Bl/6J mice (The Jackson Laboratory, Bar Harbor, ME) received either an ischemic stroke (*n* = 15) or sham surgery (*n* = 12) at twelve weeks of age (Fig. 1). More animals were allocated to the stroke group to offset expected losses from the procedure, but all animals in the stroke group survived in this study. Mice were housed individually with wetted food and hydrogel packs for four days following surgery during the acute recovery period. After this period, they were returned to their original group cages and split into either daily treadmill exercise groups (*n* = 6 sham-exercise, *n* = 8 stroke-exercise) or sedentary control groups (n = 6 sham-sedentary, *n* = 7 stroke-sedentary). Surgery and exercise groups were randomly assigned to cages. Body mass was measured twice a day for four days following surgery and weekly thereafter. Stroke recovery and sensorimotor function were assessed daily for four days following stroke and weekly thereafter. Intraosseous perfusion was measured with laser Doppler flowmetry (LDF) during the stroke or sham surgery and then weekly thereafter. After four weeks of stroke recovery with treadmill or sedentary activity, the mice were euthanized with CO_2_ asphyxiation followed by cervical dislocation. Femora and tibiae from both left (stroke-affected) and right (stroke-unaffected) hindlimbs were collected. A subset of both affected and unaffected femora from six mice (*n* = 1 sham-sedentary, *n* = 2 sham-exercise, n = 1 stroke-sedentary, n = 2 stroke-exercise) were fixed for 16 h in 5% neutral buffered formalin at 4°C and then stored in 1× phosphate buffered saline (PBS) at 4°C until assessment of osteovascular structure. All other bones were fixed for 18 h in 10% neutral buffered formalin at 4°C and then stored in 70% ethanol at 4°C.

### 2.2 Stroke procedure

Following 6–8 h of fasting, ischemic stroke was induced with the middle cerebral artery occlusion procedure using aseptic technique (Koizumi et al., 1986; Longa et al., 1989; Sakai et al., 2007). Anesthesia was induced with 5% isoflurane in a 70:30 N_2_:O_2_ gas mixture, then maintained with about 2% isoflurane. Once mice were anesthetized, fur was shaved over the three incision sites at the anterior neck (for MCAo and sham procedures), the anterior region of the temporalis muscle (for skull LDF probe), and the left proximal tibia (for bone LDF probe). Mice were placed supine on a heated pad, and rectal temperature was maintained at 37°C throughout the procedure (TCAT-2DF, Physitemp Instruments, LLC, Clifton, NJ). LDF was used to monitor both cerebral blood flow and proximal tibial perfusion using a 785-nm light source (moorVMS-LDF, Moor Instruments Ltd., Axminster, UK). For cerebral blood flow, a small, 2–5 mm-long incision was made over the right temporal bone, and a monofilament probe (VP10M200ST, Moor Instruments Ltd) was affixed directly to the skull behind the temporalis muscle with cyanoacrylate glue. Cerebral blood flow was recorded with a cutoff frequency of 15 kHz selected for the low-pass filter, along with the automatically applied 20-Hz high-pass filter. For tibial perfusion, a small, 2–5 mm-long incision was made over the proximal anteromedial side of the left tibia near the metaphysis, avoiding underlying soft tissue and muscle. A small region of the periosteum was scraped away (roughly the size of the LDF probe, or 0.5 mm^2^ circle), and a needle probe (VP4 Needle Probe, Moor Instruments Ltd) was held firmly against the bone with a micromanipulator (MM3-ALL, World Precision Instruments, Sarasota, FL). Tibial intraosseous perfusion was recorded with a cutoff frequency of 3 kHz selected for the low-pass filter and the automatic 20-Hz high-pass filter.

For the stroke or sham surgery, an incision was made over the neck midline, and the right common carotid artery (CCA), external carotid artery (ECA), and internal carotid artery (ICA) were exposed. In the sham group, saline was added to the neck incision to prevent tissue from drying out, cerebral blood flow was monitored, and tibial perfusion was recorded for 30 min. In the stroke group, temporary ligations were made to the CCA and ICA, while two permanent ligations were made to the ECA, and the vessel was cut between them. Baseline cerebral blood flow values were collected by loosening the CCA ligation for 2 min. The CCA ligation was then retightened, and a thin 6–0 nylon monofilament occluder with a silicone-coated tip (Doccol Corporation, Redlands, CA) was passed through the ECA to the ICA and MCA origin until an 80% reduction in cerebral blood flow relative to baseline was noted and maintained. The size of the silicone coating was selected based on body mass, per the manufacturer's instructions, and either a 1–2 or 2–3 mm long, 0.20–0.24 mm diameter coating was selected. Saline was added to the incision to prevent tissue from drying out. The occluding filament was left in place for 30 min, and tibial perfusion was recorded. After 30 min, the occluder was gently withdrawn, the ECA was permanently ligated, and temporary ligations were removed. For both sham and stroke procedures, the LDF probes were removed, and an intraincisional injection of bupivacaine (2 mg/kg, Marcaine, Hospira, Lake Forest, IL) was administered to the neck. The neck and skull incisions were closed with sutures (Deknatel 4-0 Non-Absorbable Silk Sutures, Teleflex, Morrisville, NC), and the hindlimb incision was closed with tissue adhesive (VetBond™, 3M Company, St. Paul, MN). Triple antibiotic ointment and 4% lidocaine cream were applied to all incision sites, and a subcutaneous injection of carprofen (7 mg/kg, Rimadyl, Zoetis, Parsippany, NJ) was administered. Subcutaneous injection of carprofen was administered again the next day and every 24 h thereafter, as needed. Bupivacaine was administered via subcutaneous injection for the first two recovery days. The severity of stroke impairments was examined weekly following surgery by assessing sensorimotor function with neuroscores (Hanne et al., 2019a).

### 2.3 Treadmill exercise

Exercise groups were acclimated to the treadmill (Exer 3/6, Columbus Instruments, Columbus, OH) for two days prior to and two days following (on recovery days 4–5) the stroke or sham procedure. After acclimation, exercise groups performed a standard moderate treadmill exercise regimen (12 m/min, 30 min, 5 days/wk, 5° incline), while sedentary groups were placed on a stationary replica treadmill for a matched time period. Stroke and sham groups performed the same exercise regimen, covering approximately 360 m.

### 2.4 Bone perfusion (tibia)

**Fig. 2:**
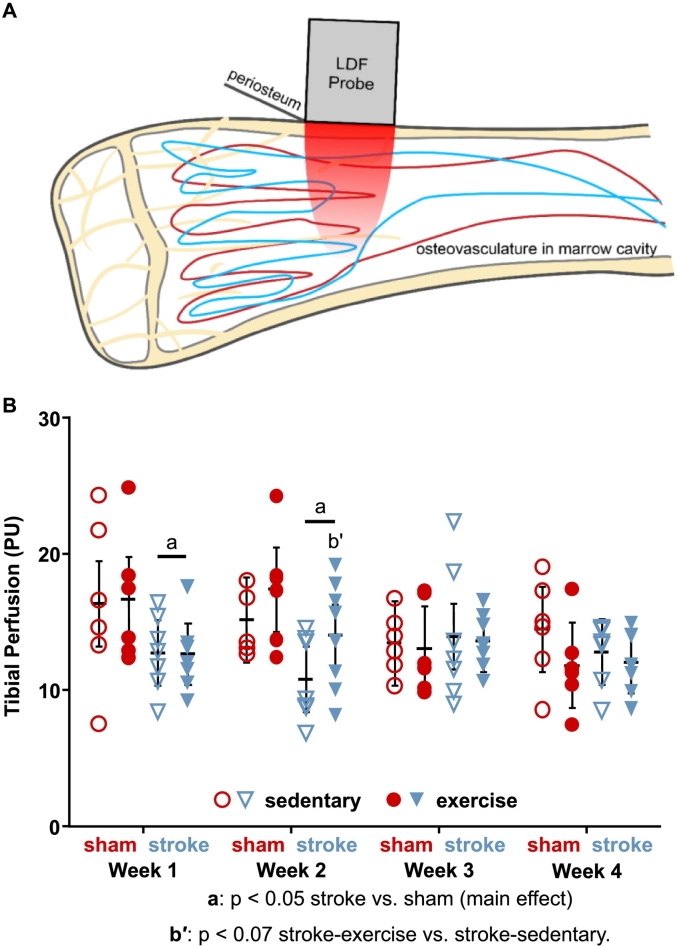
A) Intraosseous perfusion in the proximal tibia was measured serially during stroke recovery using laser Doppler flowmetry (LDF). B) Perfusion decreased in both limbs at Weeks 1 and 2 for stroke relative to sham and increased at Week 2 for exercise relative to sedentary in the stroke group (least squares mean ± 95% confidence interval across both limbs).

During the four-week recovery period, intraosseous perfusion was measured weekly in affected (left) and unaffected (right) proximal tibiae with laser Doppler flowmetry (Fig. 2A) using a modified protocol (Hanne et al., 2020) that we developed based on a previous endpoint-only procedure (Roche et al., 2013). LDF provides a functional measure of blood flow in long bones that is influenced by the amount of blood vessels, blood flow velocity, blood vessel permeability, and blood vessel size (Griffith et al., 2008). We previously showed that our modified, less invasive LDF procedure can be performed serially without inducing inflammation or gait abnormalities (Hanne et al., 2019b). After 6–8 h of fasting, anesthesia was induced with 4% isoflurane in pure oxygen and maintained with about 2% isoflurane throughout the 15- to 20-minute-long procedure. The fur over both proximal tibiae was shaved. Mice were placed supine on a heated pad, and the hindlimbs were secured with tape. Using the same methods as described above, an incision was made over one tibia, a small window of periosteum the size of the LDF probe (0.5 mm^2^) was gently removed, and the LDF needle probe was placed firmly against the bone with a micromanipulator. A 30-sec measurement was recorded, the probe was removed and repositioned, and a second 30-sec measurement was recorded. The incision was closed with tissue adhesive, and triple antibiotic ointment and lidocaine cream were applied. The procedure was repeated for the contralateral tibia. Each weekly perfusion measurement was composed of the weighted mean of the two 30-sec long measurements for that bone.

### 2.5 Osteovascular structure (femur)

Osteovascular structure was examined in the affected and unaffected femora of a subset of six mice (*n* = 1 sham-sedentary, *n* = 2 sham-exercise, n = 1 stroke-sedentary, n = 2 stroke-exercise) using contrast-enhanced micro-computed tomography (CE-CT). Each femur was cut in half transversely with a scalpel, and the distal halves were incubated with gentle shaking for 48 h at 4°C in a 3.695 mmol/L staining solution of hafnium-substituted Wells-Dawson polyoxometalate hydrate (K_16_[Hf(α_2_-P_2_W_17_O_61_)_2_]•19H_2_O) (Hf-POM) in 1 mL PBS, according to a previously described protocol (Kerckhofs et al., 2018). Samples were scanned with a phoenix nanotom® m (GE Measurement and Control Solutions, Wunstorf, Germany) using a peak X-ray tube potential of 60 kV, X-ray intensity of 140 μA, and integration time of 500 ms. A 0.1-mm-thick aluminum filter was applied to reduce beam hardening. Because of the relatively high X-ray attenuation of the Hf-POM-stained samples, a diamond-coated tungsten target was applied. Volumes were reconstructed at an isotropic voxel size of 2 μm using the GE datos|x software with a beam hardening correction setting of 7 and a Gaussian filter setting of 3.

Blood vessel and adipocyte microstructures were assessed in a manually selected volume of interest (VOI) that was 1.6-mm long (about 10% of the average femur length), starting from the proximal edge of the distal growth plate and extending proximally into the metaphysis, excluding cortical bone tissue, using the CTAn software (Bruker MicroCT, Kontich, Belgium). Within this VOI, cancellous bone was segmented, binarized via automatic 3D Otsu segmentation, and subtracted from the image, leaving the marrow volume. A manual global thresholding method was used to segment blood vessels and adipocytes. First, the adipocytes were binarized using opening (sphere with 1-voxel radius), closing (sphere with 3-voxel radius), and two despeckling operations (removes white and black speckles <650 voxels). Then the adipocyte volume fraction (adipocyte volume/marrow volume, Ad.V/Ma.V, %), adipocyte density (Ad.D, #/mm^3^), and distribution of adipocyte diameter (Ad.Di, μm) were quantified. Second, to eliminate edge artifacts due to the partial volume effect, the adipocytes were dilated (sphere with 5-voxel radius) and subtracted from the images, which made the adipocyte edges have similar grayscale values as those of the blood vessels. The images were then segmented to binarize the blood vessels using closing (sphere with 4-voxel radius), despeckling (removes continuous volumes <1050 voxels), closing (sphere with 6-voxel radius), and despeckling (removes continuous volumes <5500 voxels) steps. The vessel volume fraction (vessel volume/marrow volume, Ves.V/Ma.V, %), vessel density (Ves.D, #/mm^3^), and the distribution of vessel thickness (Ves.Th, μm) were calculated.

Using the binarized blood vessels, branching parameters of the osteovascular network were quantified using the *Skeleton 3D* and *Analyze Skeleton* plugins in BoneJ (FIJI v. 1.51n) (Doube et al., 2010; Schindelin et al., 2012), including total number of branches (e.g., vessel segments between junctions or between junctions and endpoints), number of junctions (e.g., nodes where vessels branch), number of triple points (e.g., junctions with three branches), and number of quadruple points (e.g., junctions with four branches). The distance between blood vessels and bone surfaces was calculated with custom code in MATLAB® (R2018, The MathWorks, Natick, MA). The proximity of blood vessels to bone surfaces was also examined. Similar to the procedure described above, a 1.6-mm-long VOI was manually selected in Slicer (v. 4.11.0) (Fedorov et al., 2012), starting at the proximal edge of the distal growth plate and extending proximally into the diaphysis, including the cortical bone. The VOI was exported to MATLAB®, and bone tissue, adipocytes, and blood vessels were binarized and processed using the same global threshold values and processing steps described above. The distribution of distances between blood vessel surfaces and bone surfaces (Ves.S-BS distance) was calculated using the *bwgeodesic* function in MATLAB®.

### 2.6 Osteovascular composition (tibia)

The osteovascular composition, or relative abundance of endothelial cell types in bone, was examined using confocal immunofluorescence microscopy in bone tissue sections from a subset of affected tibiae (*n* = 3 sham-sedentary, *n* = 2 sham-exercise, *n* = 5 stroke-sedentary, *n* = 4 stroke-exercise) labeled for markers of non-arterial endothelial cells (endomucin, EMCN) and endothelial cells in sinuses, arterioles, venules, and capillaries (CD31) (Ramasamy et al., 2014; Pusztaszeri et al., 2006). Endothelial cells that are positive for both EMCN and CD31 (defined as *type H*) have been shown to regulate bone-vascular crosstalk and couple osteo- and angiogenesis in long bones (Kusumbe et al., 2014). Tibiae were cut transversely at the tibiofibular junction under constant water irrigation with a low speed precision saw fitted with a diamond blade (IsoMet Low Speed Precision Cutter, Buehler, Lake Bluff, IL). The proximal halves of the tibiae were decalcified for 24 h at 4°C with constant agitation in a solution of 0.342 mol/L ethylenediaminetetraacetic acid (Fisher Scientific, Hampton, NH) diluted in 1× PBS at pH 7.6. Bone samples were then prepared for sectioning and labeling with immunofluorescent markers (Kusumbe et al., 2015). Briefly, decalcified tissue was infiltrated with a cryoprotectant solution (0.584 mol/L sucrose (S7903, Sigma-Aldrich, St. Louis, MO) and 5.556E-5 mol/L polyvinylpyrrolidone (P5288, Sigma-Aldrich) in 1× PBS) at 4°C for 24 h with constant agitation. Next, samples were incubated in an embedding medium (0.267 mol/L gelatin from porcine skin (G1890, Sigma-Aldrich), 0.584 mol/L sucrose, and 5.556E-5 mol/L polyvinylpyrrolidone in 1× PBS) at 60°C for 45 min and room temperature for 30 min and then were stored at −80°C until sectioning. In each proximal sample, 50-μm-thick longitudinal sections were obtained using a cryostat at −23°C (HN 525NX, Thermo Fisher Scientific, Waltham, MA). Sections were dried at room temperature for 30 min and then stored at −20°C.

For immunofluorescence, slides were equilibrated to room temperature for 30 min, then rehydrated in 1× PBS for 5 min. Sections were permeabilized with a solution of 4.638E-3 mol/L Triton X (T8787, Sigma-Aldrich) diluted in 1× PBS for 20 min at room temperature and blocked in a solution of donkey serum diluted 1:20 in 1× PBS for 30 min at room temperature. Sections were stained using unconjugated antibodies for EMCN (rat anti-mouse sc-65495, Santa Cruz Biotechnology, Santa Cruz, CA), CD31 (hamster anti-mouse ab119341, Abcam, Cambridge, UK), and either vascular endothelial growth factor receptor 2 (VEGFR-2, rabbit anti-mouse ab2349, Abcam) or osteocalcin (rabbit anti-mouse, ab93876, Abcam); the sections were incubated overnight at 4°C in a solution with the primary antibodies added at 1:100 to 7.728E-5 mol/L Triton X diluted in 1× PBS. Secondary antibodies (goat anti-rat with Alexa Fluor® 647, ab150159, Abcam; goat anti-hamster with Alexa Fluor® 568, A21112, Invitrogen, Carlsbad, CA; and goat anti-rabbit 488, A11034, Invitrogen) were added at 1:200 to 7.728E-5 mol/L Triton X diluted in 1× PBS, and the sections were then incubated for 60 min at room temperature. For nuclear staining, the sections were incubated for 10 min at room temperature in a 7.212E-6 mol/L solution of DAPI diluted in 1× PBS.

Immediately following staining, sections were imaged at 20× on a Zeiss Laser Scanning Microscope 880 with Airyscan (Carl Zeiss Microscopy, Thornwood, NY), and the percentage areas of CD31-positive, EMCN-positive, and type H cells, relative to total area, were quantified. Tile scan images were stitched and processed into maximum intensity projections using the microscope software (Zen 2.3 SP1, Carl Zeiss Microscopy). For each section, a region of interest equal to 10% of total tibia length was drawn in FIJI, starting at the distal edge of the proximal growth plate and extending distally into the metaphysis. CD31-positive regions inside the region of interest were manually traced into masks in FIJI. Using custom code in MATLAB®, EMCN-positive regions were automatically binarized with an adaptive threshold, and then the mask was refined with an opening (disk with 1-pixel radius) followed by a closing (disk with 2-pixel radius) operation. The CD31 mask, EMCN mask, and the intersection of both masks (type H) were calculated in FIJI. Staining for VEGFR-2 and osteocalcin was indistinguishable from background and could not be quantified. Osteovascular composition parameters were measured in two sections per bone, and the mean values were used for analysis.

### 2.7 Statistical analyses

Statistical models were analyzed with SAS (SAS University Edition v. 9.4, SAS Institute Inc., Cary, NC) with a significance level of 0.05. Statistical models were designed to determine the following: 1) acute effect of ischemic stroke on intraosseous perfusion during surgery; 2) effect of ischemic stroke and exercise on intraosseous perfusion during stroke recovery; 3) effect of stroke and exercise on osteovascular structure and composition; and 4) whether stroke differentially affects intraosseous perfusion or osteovascular structure in the affected vs. unaffected limb.

For analysis #1, the stability of the tibial perfusion measurements throughout the surgery was examined using the slope of the 30-minute LDF measurement taken during the surgical procedure. To determine if the slope for each group was different from zero, which would indicate that the perfusion measurement varied and was not stable, an unpaired *t*-test was performed for the sham group, and a Wilcoxon rank sum test was performed for the stroke group. The nonparametric test was used, because the stroke data were not normally distributed. Slopes were also compared between the stroke and sham groups with a Wilcoxon two-sample test.

For analysis #2, LDF measures of perfusion were compared between surgery and activity groups at each timepoint and limb using a mixed hierarchical linear model (procedure GLIMMIX) with interaction between all terms (Littell, 2006). Each mouse was considered a subject (replicate), assigned randomly to a group comprised of the combination of surgery and activity groups. *Surgery group* (sham or stroke) and *activity group* (sedentary or exercise) were modeled as fixed factors, *limb* as a nested within-subject factor, and *timepoint* as a longitudinal repeated measure. LDF was measured across four timepoints (Weeks 1–4) at each observational unit (each limb of each mouse). Variation among subjects within each group combination (i.e., sham-sedentary) was considered a random effect. Residuals were modeled using a compound symmetry covariance structure. A modified Kenward-Roger approximation was used to calculate denominator degrees of freedom and standard error of fixed effects (Littell, 2006). Effect differences between surgery and activity groups were compared within each timepoint (e.g., stroke-exercise vs. stroke-sedentary at Week 2) using least squares means and Tukey-Kramer adjustments for multiple comparisons (Fig. 2).

**Fig. 3:**
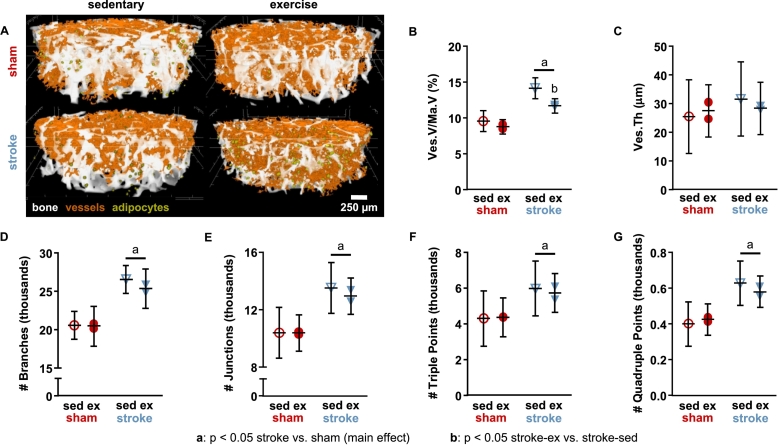
A) Osteovascular structure in the distal femoral metaphysis from contrast-enhanced micro-computed tomography. B) Blood vessel volume fraction, vessel volume (Ves.V)/marrow volume (Ma.V), was increased for stroke relative to sham, while exercise mitigated the effect of stroke. C) Mean vessel thickness (Ves.Th) was not altered by stroke or exercise. D) Vascular branching, E) number of junctions, F) number of triple points, and G) number of quadruple points were greater for stroke relative to sham. Data are least squares mean ± 95% confidence interval across affected and unaffected limbs. sed: sedentary; ex: exercise.

**Fig. 4:**
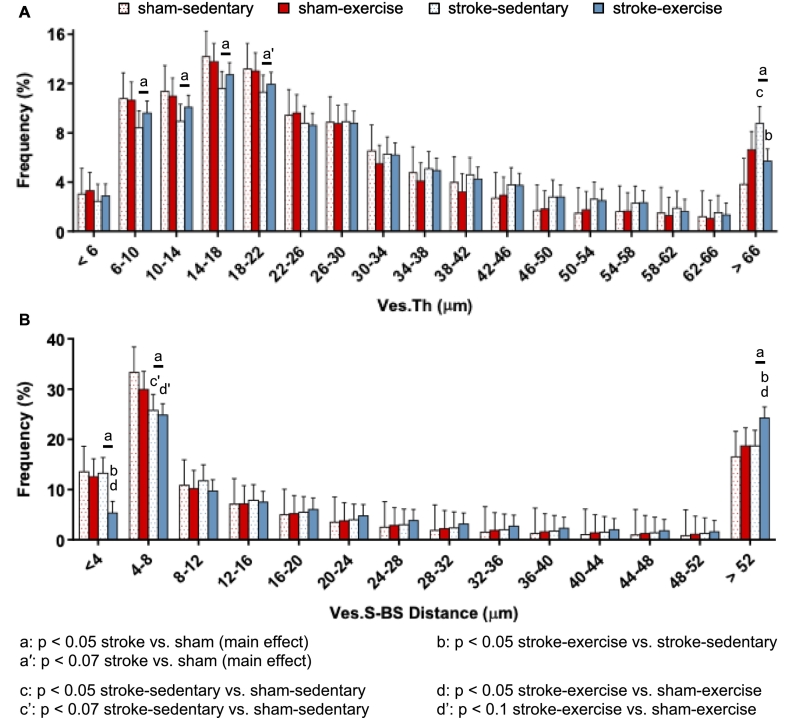
Osteovascular arrangement in the distal femoral metaphysis was quantified in a subset of mice (*n* = 1 sham-sedentary, *n* = 2 sham-exercise, n = 1 stroke-sedentary, n = 2 stroke-exercise). A) Distribution of vessel thickness (Ves.Th) was shifted post-stroke, with fewer smaller vessels and more larger vessels for stroke relative to sham. Exercise mitigated the stroke-induced increase in larger vessels. B) The relative number of blood vessels nearer to bone surfaces (smaller vessel surface to bone surface, Ves.S-BS, distance) was reduced, and the number farther from bone surfaces was increased, for both stroke (vs. sham) and exercise (vs. sedentary), especially for stroke-exercise.

**Fig. 5:**
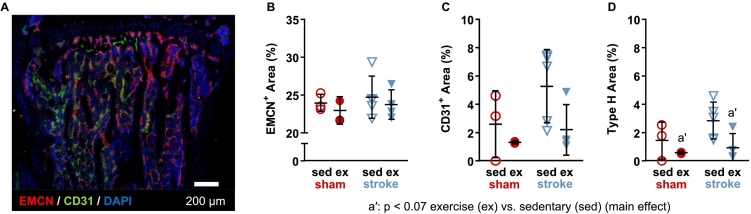
A) Representative longitudinal section in the proximal tibial metaphysis, showing osteovascular composition with immunofluorescence. B) Endomucin (EMCN)^+^ and C) CD31^+^ areas (% relative to total area) were unaffected by stroke or exercise. D) Relative area of type H endothelial cells (EMCN^+^ and CD31^+^) tended to be lower with exercise.

For analysis #3, distribution data from CE-CT (vessel thickness, blood vessel surface to bone surface distance) were compared between surgery and activity groups using a mixed hierarchical linear model (procedure GLIMMIX) with interaction between terms. The model was similar to the model used for analysis #2, except the repeated factor *week* was replaced with histogram *bins*. Variation among subjects within each group combination (e.g., sham-sedentary) was considered a random effect that was modeled with a heterogenous variance components method across each surgery group (sham or stroke). Variation among each group combination was modeled as an intercept for each group linear predictor. A modified Kenward-Roger approximation was used to calculate denominator degrees of freedom and standard error of fixed effects. Effect differences were compared between groups within histogram bins (e.g., stroke-exercise vs. stroke-sedentary within bin “a”) using least squares means with Tukey-Kramer adjustments for multiple comparisons (Fig. 4). All other CE-CT parameters (Ma.V, Ad.V/Ma.V, average Ad.Di, Ad.D, Ves.V/Ma.V, average Ves.Th, Ves.D, branches, junctions, triple points, and quadruple points) were compared between surgery and activity groups using a repeated measures factorial model (procedure MIXED) with interaction between terms. *Surgery group* and *activity group* were modeled as fixed factors, while *limb* was modeled as a repeated measure. Residual variance was modeled with compound symmetry covariance, and effect differences were compared between groups using least squares means with Tukey-Kramer adjustments for multiple comparisons (Fig. 3 and Table 1). Immunofluorescence parameters were compared between surgery and activity groups using a standard two-factor general linear model (procedure GLM) with interaction and Tukey adjustments for multiple comparisons (Fig. 5).

**Table 1:** Adipocyte microstructure in the distal femoral metaphysis.

Trait	Sham		Stroke	
	Sedentary	Exercise	Sedentary	Exercise
Number of mice	1	2	1	2
Ad.V/Ma.V (%)	0.15 ± 0.77	0.16 ± 0.55	0.15 ± 0.77	0.45 ± 0.55
Ad.D (#/mm^3^)	127.7 ± 166.4	113.5 ± 117.7	118.4 ± 166.4	237.0 ± 117.7*
Ad.Di (μm)	20.5 ± 10.3	20.7 ± 7.3	20.2 ± 10.3	23.1 ± 7.3

Least squares mean ± 95% confidence interval; *: *p* < 0.1 stroke-exercise vs. sham-exercise; Ad.V/Ma.V: adipose volume/marrow volume; Ad.D: adipose density; Ad.Di: adipose diameter.

For analysis #4, the same repeated measures models from analyses 2 and 3 were used, but least squares means were compared between limbs within surgery group (e.g., affected vs. unaffected limb within the stroke group).

LDF data during MCAo and immunofluorescence data are presented as mean ± standard deviation. Data analyzed using least squares means (repeated LDF measures, CE-CT data) are presented as least squares mean ± 95% confidence interval and are for both limbs per mouse unless otherwise noted.

## 3 Results

### 3.1 Bone perfusion

During the surgeries (cerebral occlusion for stroke, no occlusion for sham), tibial perfusion measurements remained stable throughout, with slopes that were not significantly different from zero for either stroke (−0.0063 ± 0.0415 PU/min, *p* = 0.39) or sham (0.0049 ± 0.0106 PU/min, *p* = 0.14), and these slopes were similar between the groups (*p* = 0.98). These results demonstrate that cerebral ischemia did not directly impact blood supply to the tibia during the occlusion.

During the four-week recovery period, tibial perfusion was reduced in both limbs for two weeks following stroke (Fig. 2B), with 23% lower perfusion in the stroke groups relative to the sham groups at Week 1 (*p* = 0.0064) and 24% lower perfusion at Week 2 (*p* = 0.0061), but perfusion levels were similar between surgery groups at Week 3 (*p* = 0.71) and Week 4 (*p* = 0.60). For exercise effects, at Week 2 tibial perfusion tended to be greater in stroke-exercise relative to stroke-sedentary (30%, *p* = 0.054), restoring perfusion to levels of sham-sedentary, but these effects did not occur in the sham group, with similar perfusion levels for sham-exercise and sham-sedentary (*p* = 0.32). Exercise did not affect tibial perfusion at the other timepoints (Week 1: *p* = 0.94, Week 3: *p* = 0.79, Week 4: *p* = 0.22). The transient reduction in perfusion following stroke suggests that the structure (diameter, connectivity, and amount of blood vessels) or function (vascular tone and permeability) of the osteovasculature adapted to restore blood supply, with exercise potentially hastening the recovery.

For the stroke groups, the reductions in tibial perfusion were similar in affected and unaffected limbs, with only a trend for transient differences at Week 2 (21% lower in affected vs. unaffected, *p* = 0.055) but no differences at Week 1 (*p* = 0.42), Week 3 (*p* = 0.11), or Week 4 (*p* = 0.58). Perfusion did not differ between limbs for the sham group at any timepoint (*p* = 0.61–0.90). These results suggest that post-stroke bone perfusion changes were associated more with systemic than paretic effects.

### 3.2 Osteovascular structure

Osteovascular structure was examined in the affected and unaffected femora of a subset of six mice (*n* = 1 sham-sedentary, *n* = 2 sham-exercise, n = 1 stroke-sedentary, n = 2 stroke-exercise) using contrast-enhanced micro-computed tomography (CE-CT). None of the CE-CT parameters (vessel microstructure, vessel branching, or adipocyte microstructure) differed between affected and unaffected limbs for any of the groups, which is consistent with the idea that the osteovascular changes described below may be related to systemic effects. Blood vessel volume fraction in the distal femur was 38% greater in the stroke groups than in the sham groups (*p* = 0.0061) (Fig. 3A–B). Exercise mitigated the effect of stroke, with 17% lower Ves.V/Ma.V in stroke-exercise than in stroke-sedentary (*p* = 0.027), but it did not alter Ves.V/Ma.V for sham-exercise compared to sham-sedentary (*p* = 0.20) (Fig. 3B). Group differences in vessel volume fraction were driven by differences in vessel volume, as marrow volume was similar across groups (sham-sedentary: 3.94 ± 0.55 mm^3^, sham-exercise: 4.17 ± 0.39 mm^3^, stroke-sedentary: 4.12 ± 0.55 mm^3^, stroke-exercise: 4.19 ± 0.39 mm^3^), with no significant differences between stroke and sham (*p* = 0.46) or exercise and sedentary (*p* = 0.30). Blood vessel density was variable (sham-sedentary: 54.1 ± 305.2 mm^−3^, sham-exercise: 188.2 ± 215.8 mm^−3^, stroke-sedentary: 62.2 ± 305.2 mm^−3^, stroke-exercise: 132.9 ± 215.8 mm^−3^), and mean values did not differ significantly between stroke and sham groups (*p* = 0.74) or between exercise and sedentary groups (*p* = 0.24). Mean blood vessel thickness was also similar between stroke and sham groups (*p* = 0.31) and between exercise and sedentary groups (*p* = 0.84) (Fig. 3C). The vessel network within bone was more branched following stroke, with 26% greater number of branches (*p* = 0.0089, Fig. 3D), 27% more junctions (*p* = 0.015, Fig. 3E), 33% more junctions with three branches (triple points, *p* = 0.040, Fig. 3F) and 43% more junctions with four branches (quadruple points, *p* = 0.017, Fig. 3G) for the stroke groups compared to sham groups. Exercise did not affect any of the network branching parameters.

Although the mean vessel thickness was unaltered, the distribution of Ves.Th was shifted following stroke, with fewer small-diameter vessels (6–22 μm thickness) and more larger-diameter vessels (>66 μm thickness) for stroke groups relative to sham groups (Fig. 4A). Compared to sham, the stroke groups had fewer vessels of 6–10 μm thickness (9%, *p* = 0.029), 10–14 μm thickness (8%, *p* = 0.033), and 14–18 μm thickness (8%, *p* = 0.019); tended to have fewer vessels of 18–22 μm thickness (8%, *p* = 0.056); and had more vessels of >66 μm thickness (18%, *p* = 0.010). The stroke-induced increase in larger vessels only occurred for sedentary animals, with 129% more larger vessels for stroke-sedentary relative to sham-sedentary (*p* = 0.001). Exercise mitigated this effect (*p* = 0.0032 stroke-exercise vs. stroke-sedentary), bringing vessel thickness for stroke-exercise similar to that for sham-exercise (*p* = 0.72). Taken together, ischemic stroke increased blood vessel volume fraction (Fig. 3B), increased the number of osteovascular branches (Fig. 3D–G), and increased the relative amount of larger-diameter vessels yet decreased the relative amount of small-diameter vessels (Fig. 4A). This suggests that stroke stimulated arteriogenesis, the widening of arterioles and formation of new collateral anastomoses between existing arterioles, rather than angiogenesis, the formation of new small diameter capillaries (Heil et al., 2006). Angiogenesis is stimulated by hypoxia, endocrine signaling, and inflammation, while arteriogenesis is primarily a response to shear force, blood pressure along the vessel wall, and inflammation (Heil et al., 2006). If similar changes occurred in the tibia (where perfusion was measured) as in the femur (where osteovascular structure was measured), then the rescue of intraosseous perfusion in the third week following ischemic stroke may be due in part to arteriogenesis. Though exercise should increase blood pressure and the shear forces experienced by blood vessels, exercise did not stimulate arteriogenesis in this study. One possibility is that the treadmill exercise regimen utilized is not strenuous enough to stimulate arteriogenesis – a study examining arteriogenesis in response to exercise in mice abandoned their similar treadmill training regimen in favor of free access to a running wheel for this reason (Bresler et al., 2019).

The proximity of blood vessels to bone surfaces was altered by stroke, in particular the interaction between stroke and exercise, as seen by the shifted distribution of Ves.S-BS distance (Fig. 4B). Compared to sham groups, the stroke groups had fewer blood vessels positioned within 4 μm of bone surfaces (*p* = 0.041), fewer blood vessels positioned 4–8 μm from bone surfaces (*p* = 0.0010), and more vessels positioned 52 μm or farther from bone surfaces (*p* = 0.036). These changes only occurred for stroke-exercise, producing differences relative to stroke-sedentary for distances <4 μm (59% fewer, *p* = 0.0007) and >52 μm (30% more, *p* = 0.024), as well as differences relative to sham-exercise (< 4 μm: 57% fewer, *p* = 0.0059; > 52 μm: 30% more, *p* = 0.046). For distances of 4–8 μm, the number of vessels tended to be reduced in both stroke groups, with a trend for fewer in stroke-sedentary relative to sham-sedentary (23%, *p* = 0.061), but the trend between stroke-exercise and sham-exercise was less apparent (17%, *p* = 0.080).

Adipocyte microstructure was not significantly affected by stroke or exercise, with similar values for adipocyte volume fraction, density, and diameter across groups (Table 1). Adipocyte density tended to be greater for stroke-exercise relative to sham-exercise (109%, *p* = 0.086).

### 3.3 Osteovascular composition

Vascular composition in the proximal tibial metaphysis of affected limbs (Fig. 5A) was not affected by stroke and was only marginally affected by exercise. The area of endomucin-expressing cells, relative to total area, was similar between stroke and sham groups (*p* = 0.43) and between exercise and sedentary groups (*p* = 0.54) (Fig. 5B). Similarly, the relative CD31-expressing cell area was also similar between stroke and sham groups (*p* = 0.18) and exercise and sedentary groups (*p* = 0.11) (Fig. 5C). The relative area of type H cells expressing both EMCN and CD31 was not significantly different between stroke and sham groups (*p* = 0.20) but tended to be lower for exercise compared to sedentary groups (186%, *p* = 0.059) (Fig. 5D). Since increased endomucin expression would be indicative of angiogenesis (Park-Windhol et al., 2017), the lack of change in endomucin-expressing endothelium further supports that ischemic stroke stimulated arteriogenesis rather than angiogenesis. These data show a trend suggestive of more CD31-expressing endothelium in response to ischemic stroke, but more samples are needed to determine if this effect is real.

## 4 Discussion

This study is the first to report the effects of ischemic stroke on osteovasculature. Middle cerebral artery occlusion in mice was associated with increased blood vessel volume within the distal femoral metaphysis, primarily due to increased vascular branching and a greater relative amount of larger-diameter vessels. These changes assessed after four weeks were preceded by stroke-related decreases in osseous perfusion measured in the proximal tibia during the first two weeks of recovery. The stroke-related changes in the proximity of blood vessels to bone surfaces were exacerbated by stroke-exercise interactions, with fewer vessels near to bone surfaces. Because previous studies have shown that active sites of remodeling are associated with an increased concentration of capillaries within 50 μm of the remodeling surface compared to non-remodeling bone surfaces (Kristensen et al., 2013), these changes to osteovascular structure and function may reflect a reduced osteogenic capacity that could play a role in bone loss in stroke patients. A major limitation of this study is that only a small subset of samples were used to quantify changes to osteovascular structure – the reported differences are interesting observations that warrant further study.

Limb disuse via hindlimb suspension has also been shown to decrease bone perfusion in rodents (Stabley et al., 2013; Colleran et al., 2000). However, in our study, mice remained ambulatory following the mild-to-moderate stroke severity, and we previously showed that this cohort had no alterations in limb coordination patterns during gait (Hanne et al., 2019a). In addition, the reduced perfusion was present even in the stroke-exercise group during early recovery. Together, these results suggest that the osteovascular changes in this study result primarily from more systemic stroke effects rather than from limb disuse. Future work with this induced stroke model may improve understanding about the pathogenesis of skeletal fragility in stroke patients, in particular examining how osteovascular structure and function may contribute. In our previous paper examining material composition of the tibial midshaft in these same mice we found that stroke tended to decrease mineral-to-matrix ratio, which has been shown to be correlated with stiffness (Akkus et al., 2004), in the stroke-sedentary but not stroke-exercise groups (Hanne et al., 2019a). A future study should perform mechanical testing on the hindlimb bones to determine if stroke does increase skeletal fragility, and if exercise can rescue the effect.

In this study, stroke decreased functional blood supply within the proximal tibia for two weeks. Perfusion was reduced in both affected and unaffected limbs during the first two weeks of recovery, and tended to be lower in the affected limb than in the unaffected limb during the second week of recovery, mimicking the differential decreases in lower leg blood flow observed in previous studies of chronic stroke patients with mild-to-moderate gait asymmetry (Ivey et al., 2004, Ivey et al., 2010). The decreased perfusion post-stroke was transient, returning to sham levels by the third week of recovery in all stroke groups. Exercise accelerated this recovery, with perfusion in the stroke-exercise group returning to sham-sedentary levels by the second week, indicating that moderate exercise may be a viable strategy for increasing intraosseous blood supply following stroke. Although a number of studies in human stroke patients have shown increased limb perfusion with exercise following stroke (Ivey et al., 2010; Billinger et al., 2009), this study is the first to demonstrate that exercise restores perfusion for the microvasculature within bone, as well. A potential limitation of the repeated perfusion measurements is that a small amount of periosteum is removed from the tibiae each week. Because periosteum is well vascularized and an important stem cell niche for bone tissue, disrupting it each week could affect bone and osteovascular outcomes. Potential confounding effects are mitigated somewhat by the similar amount of periosteal disruption across groups for LDF measurements.

Perfusion measurements alone do not provide information about changes to the structure or composition of the vascular network, since reduced perfusion could reflect decreased vascular density, vessel diameter, vascular permeability, or blood velocity or increased vascular tone. Ischemic stroke resulted in increased osteovascular volume and branching in the distal femur by the end of the four-week recovery period. Considering the early transient reductions in bone perfusion in the proximal tibia, the increased osteovascular volume may be a compensatory response to offset perfusion deficits. Ischemic stroke also increased the relative amount of thicker blood vessels in the distal femur, which could indicate arteriogenesis, the widening of existing vessels in response to inflammation and fluid shear (Heil et al., 2006). However, a study examining the progression of these changes over time, and at the same site, is needed to assess the relative timing and relationship between them.

Ischemic stroke is associated with many conditions that could contribute to the reduced intraosseous perfusion observed, including systemic changes to hemodynamics, increased vascular tone, increased vascular resistance, or decreased vasodilation. Vascular tone, which constricts blood vessels and decreases perfusion, was higher in cutaneous blood vessels in the paretic hands of patients with ischemic lesions (Herbaut et al., 1990) and in denervated arteries in rabbit ears (Bevan et al., 1993), suggesting that stroke-related damage to the central nervous system could contribute to the reduced intraosseous perfusion in our study. Stroke has been associated with increased vascular elasticity (a measure of vascular resistance) in the forearm (Pang et al., 2013), and resistance arteries in cutaneous blood vessels in the paretic arms of stroke patients were also less responsive to exogenous acetylcholine-induced vasodilation (Wang et al., 2002), which would increase vascular resistance. However, the effect of stroke on the arteries that supply bone are unknown, and future studies are required to determine whether increased vessel volume and branching are compensatory for changes to vascular tone, resistance, or sensitivity to vasodilators following stroke within long bones. Exercise restored the stroke-related perfusion deficits by the second week of recovery and reduced the osteovascular volume gains relative to stroke-sedentary. In previous studies, moderate aerobic endurance training decreased peripheral vascular resistance in rats (Karhunen et al., 1988) and increased vasodilator bioavailability in the leg in humans (Sugawara et al., 2007), suggesting that the exercise-related perfusion increases in our study may result, at least in part, from improved vascular function. Although not statistically significant, exercise appeared to mitigate the effects of stroke in blood vessel thickness distribution (i.e., the reduced number of small-diameter vessels and increased number of larger-diameter vessels), which should be explored further in future studies. Interestingly, exercise mitigated the stroke-related increases in intraosseous vessel volume, further supporting the idea that the increased vessel volume could be a pathological compensatory response and does not necessarily reflect improved vascular function.

Although stroke increased the volume of blood vessels in bone, the structure and composition of the osteovasculature were also affected, which may contribute to bone loss following stroke. The combination of stroke and exercise decreased the relative number of blood vessels near bone surfaces, which have been shown to be associated with sites of active bone remodeling (Kristensen et al., 2013; Prisby et al., 2011; Andersen et al., 2009). Exercise, in mitigating increased blood vessel volume post-stroke, may have also reduced the number of branches in close contact with bone surfaces. Since exercise alone did not impact vessel-to-bone proximity (i.e., in sham-exercise vs. sham-sedentary), and since stroke-exercise exhibited the lowest number of blood vessels closest to bone surfaces (<4 μm) and largest number of vessels farthest from bone surfaces (>66 μm), exercise may have negative consequences for this metric post-stroke. Despite the increased amount of osteovasculature, the reduction in the relative number of vessels in close proximity to bone, resulting primarily from loss of small vessels, may explain the lack of exercise-induced gains in bone microstructure following stroke, which we found in the same region using the same bones as in this study (Hanne et al., 2019a).

Although stroke did not significantly affect the relative area of type H cells in the tibia, it did shift the distribution of blood vessel diameter in the femur, decreasing the number of vessels between 6 and 22 μm thickness (a measure of diameter), which matches the reported diameters of columnar and arched type H capillaries (5–10 μm and 15–20 μm diameter, respectively) known to regulate osteogenesis (Ramasamy et al., 2014; Kusumbe et al., 2014). Stroke causes chronic inflammation (Lambertsen et al., 2012), which stimulates angiogenesis and arteriogenesis (Heil et al., 2006; Costa et al., 2007) and thus may be responsible for the increased vascular volume and branching following stroke, but inflammation and hypoxia also dysregulate Notch signaling (Quillard et al., 2010), the mechanism by which type H capillaries regulate bone modeling (Ramasamy et al., 2014; Kusumbe et al., 2014). Conversely, exercise tended to lower the relative area of type H cells in the proximal tibia in both sham and stroke groups but did not affect the number of branches or vessel thickness distribution in the distal femur. The reduction in relative area of type H cells in the exercise groups may be due to increased hypoxia during exercise if perfusion has been negatively affected. In future studies, weekly serum assays for markers of angiogenesis, inflammation, and bone remodeling may help elucidate the interaction between bone and vascular tissue following exercise and stroke interventions.

The effects of marrow adipose on osteovasculature are not well understood but could play a role in bone loss following stroke. In this study, neither stroke nor exercise affected the relative volume of adipocytes in the distal femur. However, as adipose grows it secretes inflammatory cytokines that stimulate macrophage activity and angiogenesis to aid its expansion (Crandall et al., 1997; Christiaens and Lijnen, 2006; Weisberg et al., 2003), which could also interfere with type H activity. Further research is needed to determine the interaction between marrow adipose, intraosseous perfusion, exercise, and skeletal integrity. Again, serum measurements of circulating factors may elucidate these interactions.

Finally, the MCAo ischemic stroke procedure used here, while well-established as a model to mimic human stroke, does have potential limitations. In this study, a thin filament was passed through the external carotid artery to temporarily occlude blood flow in the right middle cerebral artery and then removed to allow reperfusion. This occlusion-reperfusion mimics the most common type of ischemic stroke condition observed in human patients (Benjamin et al., 2019). The technique used in our study leaves the ECA permanently ligated. Ligating the ECA does not cause infarctions in the brain (Longa et al., 1989) but impedes blood supply to facial muscles including the masticator muscles, which could negatively impact feeding and bodyweight (Dittmar et al., 2003). An alternative MCAo procedure involves passing the occluding filament through the common carotid artery and then repairing the CCA ligation after the occluder is removed (CCA-repair MCAo), leaving no permanent occlusions (Dittmar et al., 2003; Hu et al., 2023). In rats the CCA-repair MCAo surgery caused less weight loss for two days following surgery compared to ECA-ligation MCAo (Dittmar et al., 2003), while in mice no differences in body mass were observed (Hu et al., 2023). However, in all MCAo studies, including ours, mice that undergo MCAo lose significant body mass compared to the sham groups (Hanne et al., 2019a; Dittmar et al., 2003; Hu et al., 2023). Since weight loss has been associated with bone mineral density loss in humans (Holbrook and Barrett-Connor, 1993), future studies examining bone may consider using an alternative MCAo procedure or have additional controls to help account for this effect.

In this study, we extended previous clinical findings that limb perfusion is reduced following ischemic stroke and demonstrated for the first time, using a mouse model, that intraosseous perfusion is also reduced during early stroke recovery and notably even in the absence of limb disuse. These functional deficits occurred in conjunction with observed changes in osteovascular structure, including potentially compensatory increases in vascular volume, branching, and the number of larger vessels. Further, although exercise mitigated the negative effects of stroke on bone perfusion and vessel volume, the combination of stroke and exercise altered the vessel proximity to bone to a less osteogenic arrangement with fewer small vessels located near bone. These findings suggest that exercise like this moderate treadmill regime may not be beneficial for osteovascular structure, at least if initiated during early recovery, although more studies are needed to examine if these effects extend to other exercise therapies or rehabilitation strategies. Examining potential mechanisms for these changes in osteovascular structure and function following stroke may provide insight for mitigating skeletal fragility in human stroke patients.

## CRediT authorship contribution statement

**Nicholas J. Hanne:** Writing – review & editing, Writing – original draft, Methodology, Investigation, Formal analysis, Conceptualization. **Andrew J. Steward:** Writing – review & editing, Methodology, Investigation, Formal analysis, Conceptualization. **Carla Geeroms:** Writing – review & editing, Validation, Methodology, Formal analysis. **Elizabeth D. Easter:** Writing – review & editing, Validation, Methodology, Investigation. **Hannah T. Gensch:** Writing – review & editing, Methodology, Investigation, Formal analysis. **Greet Kerckhofs:** Writing – review & editing, Writing – original draft, Validation, Resources, Methodology, Investigation, Formal analysis, Conceptualization. **Tatjana N. Parac-Vogt:** Writing – review & editing, Resources, Methodology, Conceptualization. **Huaxin Sheng:** Writing – review & editing, Methodology, Conceptualization. **Jacqueline H. Cole:** Writing – review & editing, Supervision, Resources, Project administration, Methodology, Funding acquisition, Conceptualization.

## Funding

This work was funded by the 10.13039/100000002National Institutes of Health [grant number K12HD073945], the 10.13039/100000968American Heart Association [grant number 17GRNT33710007], and the American Society of Biomechanics [Junior Faculty Research Award]. The content is solely the responsibility of the authors and does not necessarily represent the official views of the NIH.

## Declaration of competing interest

The authors have no conflicts of interests to declare.

## Data Availability

Data will be made available on request.

## References

[bb0005] Akkus O., Adar F., Schaffler M.B. (2004). Age-related changes in physicochemical properties of mineral crystals are related to impaired mechanical function of cortical bone. Bone.

[bb0010] Andersen T.L., Sondergaard T.E., Skorzynska K.E., Dagnaes-Hansen F., Plesner T.L., Hauge E.M., Plesner T., Delaisse J.-M. (2009). A physical mechanism for coupling bone resorption and formation in adult human bone. Am. J. Pathol..

[bb0015] Beaupré G.S., Lew H.L. (2006). Bone-density changes after stroke. Am. J. Phys. Med. Rehabil..

[bb0020] Benjamin E.J., Muntner P., Alonso A., Bittencourt M.S., Callaway C.W., Carson A.P., Chamberlain A.M., Chang A.R., Cheng S., Das S.R., Delling F.N., Djousse L., Elkind M.S.V., Ferguson J.F., Fornage M., Jordan L.C., Khan S.S., Kissela B.M., Knutson K.L., Kwan T.W., Lackland D.T., Lewis T.T., Lichtman J.H., Longenecker C.T., Loop M.S., Lutsey P.L., Martin S.S., Matsushita K., Moran A.E., Mussolino M.E., O’Flaherty M., Pandey A., Perak A.M., Rosamond W.D., Roth G.A., Sampson U.K.A., Satou G.M., Schroeder E.B., Shah S.H., Spartano N.L., Stokes A., Tirschwell D.L., Tsao C.W., Turakhia M.P., VanWagner L.B., Wilkins J.T., Wong S.S., Virani S.S. (2019). American Heart Association Council on Epidemiology and Prevention Statistics Committee and Stroke Statistics Subcommittee, heart disease and stroke statistics-2019 update: a report from the American Heart Association. Circulation.

[bb0025] Bevan R.D., Clementson A., Joyce E., Bevan J.A. (1993). Sympathetic denervation of resistance arteries increases contraction and decreases relaxation to flow. Am. J. Physiol..

[bb0030] Biffar A., Dietrich O., Sourbron S., Duerr H.-R., Reiser M.F., Baur-Melnyk A. (2010). Diffusion and perfusion imaging of bone marrow. Eur. J. Radiol..

[bb0035] Billinger S.A., Gajewski B.J., Guo L.X., Kluding P.M. (2009). Single limb exercise induces femoral artery remodeling and improves blood flow in the hemiparetic leg poststroke. Stroke.

[bb0040] Borschmann K., Pang M.Y.C., Iuliano S., Churilov L., Brodtmann A., Ekinci E.I., Bernhardt J. (2015). Changes to volumetric bone mineral density and bone strength after stroke: a prospective study. Int. J. Stroke.

[bb0045] Bresler A., Vogel J., Niederer D., Gray D., Schmitz-Rixen T., Troidl K. (2019). Development of an exercise training protocol to investigate arteriogenesis in a murine model of peripheral artery disease. Int. J. Mol. Sci..

[bb0050] Christiaens V., Lijnen H.R. (2006). Role of the fibrinolytic and matrix metalloproteinase systems in development of adipose tissue. Arch. Physiol. Biochem..

[bb0055] Colleran P.N., Wilkerson M.K., Bloomfield S.A., Suva L.J., Turner R.T., Delp M.D. (2000). Alterations in skeletal perfusion with simulated microgravity: a possible mechanism for bone remodeling. J. Appl. Physiol..

[bb0060] Costa C., Incio J., Soares R. (2007). Angiogenesis and chronic inflammation: cause or consequence?. Angiogenesis.

[bb0065] Crandall D.L., Hausman G.J., Kral J.G. (1997). A review of the microcirculation of adipose tissue: anatomic, metabolic, and angiogenic perspectives. Microcirculation.

[bb0070] Dittmar M., Spruss T., Schuierer G., Horn M. (2003). External carotid artery territory ischemia impairs outcome in the endovascular filament model of middle cerebral artery occlusion in rats. Stroke.

[bb0075] Doube M., Kłosowski M.M., Arganda-Carreras I., Cordelières F.P., Dougherty R.P., Jackson J.S., Schmid B., Hutchinson J.R., Shefelbine S.J. (2010). BoneJ: free and extensible bone image analysis in ImageJ. Bone.

[bb0080] Eng J.J., Pang M.Y.C., Ashe M.C. (2008). Balance, falls, and bone health : role of exercise in reducing fracture risk after stroke. J. Rehabil. Res. Dev..

[bb0085] Fedorov A., Beichel R., Kalpathy-Cramer J., Finet J., Fillion-Robin J.-C., Pujol S., Bauer C., Jennings D., Fennessy F., Sonka M., Buatti J., Aylward S., Miller J.V., Pieper S., Kikinis R. (2012). 3D Slicer as an image computing platform for the quantitative imaging network. Magn. Reson. Imaging.

[bb0090] Fisher A., Srikusalanukul W., Davis M., Smith P. (2013). Poststroke hip fracture: prevalence, clinical characteristics, mineral-bone metabolism, outcomes, and gaps in prevention. Stroke Res. Treat..

[bb0095] Frost H.M. (2003). Bone's mechanostat: a 2003 update. Anat. Rec. A Discov. Mol. Cell. Evol. Biol..

[bb0100] Griffith J.F., Yeung D.K.W., Antonio G.E., Lee F.K.H., Hong A.W.L., Wong S.Y.S., Lau E.M.C., Leung P.C. (2005). Vertebral bone mineral density, marrow perfusion, and fat content in healthy men and men with osteoporosis: dynamic contrast-enhanced MR imaging and MR spectroscopy. Radiology.

[bb0105] Griffith J.F., Yeung D.K.W., Antonio G.E., Wong S.Y.S., Kwok T.C.Y., Woo J., Leung P.C. (2006). Vertebral marrow fat content and diffusion and perfusion indexes in women with varying bone density: MR evaluation. Radiology.

[bb0110] Griffith J.F., Yeung D.K., Tsang P.H., Choi K.C., Kwok T.C., Ahuja A.T., Leung K.S., Leung P.C. (2008). Compromised bone marrow perfusion in osteoporosis. J. Bone Miner. Res..

[bb0115] Hanne N.J., Steward A.J., Sessions M.R., Thornburg H.L., Sheng H., Cole J.H. (2019). Stroke prevents exercise-induced gains in bone microstructure but not composition in mice. J. Biomech. Eng..

[bb0125] Hanne N.J., Easter E.D., Cole J.H. (2019). Minimally invasive laser Doppler flowmetry is suitable for serial bone perfusion measurements in mice. Bone Rep..

[bb0120] Hanne N.J., Easter E.D., Stangeland-Molo S., Cole J.H. (2020). A minimally invasive technique for serial intraosseous perfusion measurements in the murine tibia using laser Doppler flowmetry. MethodsX.

[bb0130] Heil M., Eitenmüller I., Schmitz-Rixen T., Schaper W. (2006). Arteriogenesis versus angiogenesis: similarities and differences. J. Cell. Mol. Med..

[bb0135] Herbaut A.G., Cole J.D., Sedgwick E.M. (1990). A cerebral hemisphere influence on cutaneous vasomotor reflexes in humans. J. Neurol. Neurosurg. Psychiatry.

[bb0140] Holbrook T.L., Barrett-Connor E. (1993). The association of lifetime weight and weight control patterns with bone mineral density in an adult community. Bone Miner..

[bb0145] Hu Y., Yang Z.-H., Yan F., Huang S.-F., Wang R.-L., Han Z.-P., Fan J.-F., Zheng Y.-M., Liu P., Luo Y.-M., Li S.-J. (2023). CCA repair or ECA ligation—which middle cerebral artery occlusion is better in the reperfusion mouse model?. Ibrain.

[bb0150] Ivey F.M., Gardner A.W., Dobrovolny C.L., Macko R.F. (2004). Unilateral impairment of leg blood flow in chronic stroke patients. Cerebrovasc. Dis..

[bb0155] Ivey F.M., Hafer-Macko C.E., Ryan A.S., Macko R.F. (2010). Impaired leg vasodilatory function after stroke: adaptations with treadmill exercise training. Stroke.

[bb0160] Jones J.P.J.M.D. (1993). Fat embolism, intravascular coagulation, and osteonecrosis. Clin. Orthop..

[bb0165] Jørgensen L., Jacobsen B.K. (2001). Functional status of the paretic arm affects the loss of bone mineral in the proximal humerus after stroke: a 1-year prospective study. Calcif. Tissue Int..

[bb0170] Jørgensen L., Jacobsen B.K., Wilsgaard T., Magnus J.H. (2000). Walking after stroke: does it matter? Changes in bone mineral density within the first 12 months after stroke. A longitudinal study. Osteoporos. Int..

[bb0175] Kanis J., Oden A., Johnell O. (2001). Acute and long-term increase in fracture risk after hospitalization for stroke. Stroke.

[bb0180] Karhunen M.K., Rämö M.P., Kettunen R., Hirvonen L. (1988). The cardiovascular effects of deconditioning after endurance training in rats. Acta Physiol. Scand..

[bb0185] Kerckhofs G., Stegen S., van Gastel N., Sap A., Falgayrac G., Penel G., Durand M., Luyten F.P., Geris L., Vandamme K., Parac-Vogt T., Carmeliet G. (2018). Simultaneous three-dimensional visualization of mineralized and soft skeletal tissues by a novel microCT contrast agent with polyoxometalate structure. Biomaterials.

[bb0190] Koizumi J., Yoshida Y., Nakazawa T., Ooneda G. (1986). Experimental studies of ischemic brain edema, I: a new experimental model of cerebral embolism in rats in which recirculation can be introduced in the ischemic area. Jpn. J. Stroke.

[bb0195] Kristensen H.B., Andersen T.L., Marcussen N., Rolighed L., Delaisse J.-M. (2013). Increased presence of capillaries next to remodeling sites in adult human cancellous bone. J. Bone Miner. Res..

[bb0200] Kusumbe A.P., Ramasamy S.K., Adams R.H. (2014). Coupling of angiogenesis and osteogenesis by a specific vessel subtype in bone. Nature.

[bb0205] Kusumbe A.P., Ramasamy S.K., Starsichova A., Adams R.H. (2015). Sample preparation for high-resolution 3D confocal imaging of mouse skeletal tissue. Nat. Protoc..

[bb0210] Lambertsen K.L., Biber K., Finsen B. (2012). Inflammatory cytokines in experimental and human stroke. J. Cereb. Blood Flow Metab..

[bb0215] Laroche M. (2002). Intraosseous circulation from physiology to disease. Joint Bone Spine.

[bb0220] Leblanc A.D., Schneider V.S., Evans H.J., Engelbretson D.A., Krebs J.M. (1990). Bone mineral loss and recovery after 17 weeks of bed rest. J. Bone Miner. Res..

[bb0225] R.C. Littell, ed., SAS for mixed models, 2nd ed, SAS Institute, Inc., Cary, N.C, 2006.

[bb0230] Longa E.Z., Weinstein P.R., Carlson S., Cummins R. (1989). Reversible middle cerebral artery occlusion without craniectomy in rats. Stroke.

[bb0235] McCabe L.R., Irwin R., Tekalur A., Evans C., Schepper J.D., Parameswaran N., Ciancio M. (2019). Exercise prevents high fat diet-induced bone loss, marrow adiposity and dysbiosis in male mice. Bone.

[bb0240] Owen-Woods C., Kusumbe A. (2022). Fundamentals of bone vasculature: specialization, interactions and functions. Semin. Cell Dev. Biol..

[bb0245] Pang M.Y.C., Lau R.W.K. (2010). The effects of treadmill exercise training on hip bone density and tibial bone geometry in stroke survivors: a pilot study. Neurorehabil. Neural Repair.

[bb0250] Pang M.Y.C., Yang F.Z.H., Jones A.Y.M. (2013). Vascular elasticity and grip strength are associated with bone health of the hemiparetic radius in people with chronic stroke: implications for rehabilitation. Phys. Ther..

[bb0255] Park-Windhol C., Ng Y.S., Yang J., Primo V., Saint-Geniez M., D’Amore P.A. (2017). Endomucin inhibits VEGF-induced endothelial cell migration, growth, and morphogenesis by modulating VEGFR2 signaling. Sci. Rep..

[bb0260] Peppen R.P.V., Kwakkel G., Wood-Dauphinee S., Hendriks H.J., der Wees P.J.V., Dekker J. (2004). The impact of physical therapy on functional outcomes after stroke: what’s the evidence?. Clin. Rehabil..

[bb0265] Prisby R., Guignandon A., Vanden-Bossche A., Mac-Way F., Linossier M.-T., Thomas M., Laroche N., Malaval L., Langer M., Peter Z.-A., Peyrin F., Vico L., Lafage-Proust M.-H. (2011). Intermittent PTH(1–84) is osteoanabolic but not osteoangiogenic and relocates bone marrow blood vessels closer to bone-forming sites. J. Bone Miner. Res..

[bb0270] Pusztaszeri M.P., Seelentag W., Bosman F.T. (2006). Immunohistochemical expression of endothelial markers CD31, CD34, von Willebrand factor, and Fli-1 in normal human tissues. J. Histochem. Cytochem..

[bb0275] Quillard T., Devallière J., Coupel S., Charreau B. (2010). Inflammation dysregulates Notch signaling in endothelial cells: implication of Notch2 and Notch4 to endothelial dysfunction. Biochem. Pharmacol..

[bb0280] Ramasamy S.K., Kusumbe A.P., Wang L., Adams R.H. (2014). Endothelial Notch activity promotes angiogenesis and osteogenesis in bone. Nature.

[bb0285] Ramnemark A., Nyberg L., Borssén B., Olsson T., Gustafson Y. (1998). Fractures after stroke. Osteoporos. Int..

[bb0290] Ramnemark A., Nyberg L., Lorentzon R., Englund U., Gustafson Y. (1999). Progressive hemiosteoporosis on the paretic side and increased bone mineral density in the nonparetic arm the first year after severe stroke. Osteoporos. Int..

[bb0295] Reeve J., Arlot M., Wootton R., Edouard C., Tellez M., Hesp R., Green J.R., Meunier P.J. (1988). Skeletal blood flow, iliac histomorphometry, and strontium kinetics in osteoporosis: a relationship between blood flow and corrected apposition rate. J. Clin. Endocrinol. Metab..

[bb0300] Roche B., Vanden-Bossche A., Normand M., Malaval L., Vico L., Lafage-Proust M.H. (2013). Validated laser Doppler protocol for measurement of mouse bone blood perfusion — response to age or ovariectomy differs with genetic background. Bone.

[bb0305] Sakai H., Sheng H., Yates R.B., Ishida K., Pearlstein R.D., Warner D.S. (2007). Isoflurane provides long-term protection against focal cerebral ischemia in the rat. Anesthesiology.

[bb0310] Schindelin J., Arganda-Carreras I., Frise E., Kaynig V., Longair M., Pietzsch T., Preibisch S., Rueden C., Saalfeld S., Schmid B., Tinevez J.-Y., White D.J., Hartenstein V., Eliceiri K., Tomancak P., Cardona A. (2012). Fiji: an open-source platform for biological-image analysis. Nat. Methods.

[bb0315] Stabley J.N., Prisby R.D., Behnke B.J., Delp M.D. (2013). Chronic skeletal unloading of the rat femur: mechanisms and functional consequences of vascular remodeling. Bone.

[bb0320] Stegen S., Carmeliet G. (2018). The skeletal vascular system – breathing life into bone tissue. Bone.

[bb0325] Styner M., Thompson W.R., Galior K., Uzer G., Wu X., Kadari S., Case N., Xie Z., Sen B., Romaine A., Pagnotti G.M., Rubin C.T., Styner M.A., Horowitz M.C., Rubin J. (2014). Bone marrow fat accumulation accelerated by high fat diet is suppressed by exercise. Bone.

[bb0330] Styner M., Pagnotti G.M., McGrath C., Wu X., Sen B., Uzer G., Xie Z., Zong X., Styner M.A., Rubin C.T., Rubin J. (2017). Exercise decreases marrow adipose tissue through ß-oxidation in obese running mice. J. Bone Miner. Res..

[bb0335] Sugawara J., Komine H., Hayashi K., Yoshizawa M., Otsuki T., Shimojo N., Miyauchi T., Yokoi T., Maeda S., Tanaka H. (2007). Systemic α-adrenergic and nitric oxide inhibition on basal limb blood flow: effects of endurance training in middle-aged and older adults. Am. J. Physiol. Heart Circ. Physiol..

[bb0340] Vico L., Collet P., Guignandon A., Lafage-Proust M.-H., Thomas T., Rehailia M., Alexandre C. (2000). Effects of long-term microgravity exposure on cancellous and cortical weight-bearing bones of cosmonauts. Lancet.

[bb0345] Wang J.S., Yang C.F., Liaw M.Y., Wong M.K. (2002). Suppressed cutaneous endothelial vascular control and hemodynamic changes in paretic extremities with edema in the extremities of patients with hemiplegia. Arch. Phys. Med. Rehabil..

[bb0350] Weatherholt A.M., Avin K.G., Hurd A.L., Cox J.L., Marberry S.T., Santoni B.G., Warden S.J. (2015). Peripheral quantitative computed tomography (pQCT) predicts humeral diaphysis torsional mechanical properties with good short-term precision. J. Clin. Densitom..

[bb0355] Weisberg S.P., McCann D., Desai M., Rosenbaum M., Leibel R.L., Ferrante A.W. (2003). Obesity is associated with macrophage accumulation in adipose tissue. J. Clin. Invest..

